# Development of a Charting Method to Monitor the Individual Performance of Surgeons at the Beginning of Their Career

**DOI:** 10.1371/journal.pone.0041944

**Published:** 2012-07-31

**Authors:** Antoine Duclos, Matthew J. Carty, Jean-Louis Peix, Cyrille Colin, Stuart R. Lipsitz, Jean-Louis Kraimps, Fabrice Menegaux, François Pattou, Fréderic Sebag, Nicolas Voirin, Sandrine Touzet, Stéphanie Bourdy, Jean-Christophe Lifante, CATHY Study Group, Matthew Carty, Stuart Lipsitz, Laurent Arnalsteen, Robert Caizzo, Bruno Carnaille, Guelareh Dezfoulian, Carole Eberle, Ziad El Khatib, Emmanuel Fernandez, Antoine Lamblin, François Pattou, Marie-France Six, Stéphanie Bourdy, Laetitia Bouveret, Cyrille Colin, Antoine Duclos, Guibert Guibert, Marie-Annick Le Pogam, Jean-Christophe Lifante, Jean-Louis Peix, Gaétan Singier, Pietro Soardo, Sandrine Touzet, Nicolas Voirin, Pascal Auquier, Jean-François Henry, Claire Morando, Frédéric Sebag, Sam Van Slycke, Ines Akrout, Fares Benmiloud, Jean-Paul Chigot, Isabelle Colombet, Ga≑lle Godiris-Petit, Pierre Leyre, Fabrice Ménégaux, Séverine Noullet, Benoıˆt Royer, Christophe Tresallet, Thibault Desurmont, Claudia Dominguez, Jean-Louis Kraimps, Chiara Odasso, Laetitia Rouleau, Yves-Louis Chapuis, Pierre Durieux, Alain Lepape, Frédéric Triponez

**Affiliations:** Boston; Lille; Lyon; Marseille; Paris; Poitiers; Scientific Committee; 1 Hospices Civils de Lyon, Pôle Information Médicale Evaluation Recherche, Lyon, France; Université de Lyon, EA Santé-Individu-Société 4129, Lyon, France; 2 Center for Surgery and Public Health, Brigham and Women’s Hospital - Harvard Medical School, Boston, Massachusetts, United States of America; 3 Hospices Civils de Lyon, Centre Hospitalier Lyon Sud, Service de Chirurgie Générale et Endocrinienne, Pierre Bénite, France; 4 Department of Endocrine Surgery, Poitiers University, Jean Bernard Hospital, Poitiers, France; 5 Assistance Publique - Hôpitaux de Paris, Hôpital la Pitié-Salpêtrière, Service de Chirurgie Générale, Viscérale et Endocrinienne, Paris, France; 6 CHRU de Lille, Chirurgie Générale et Endocrinienne, Lille, France; Université Lille nord de France, INSERM, UMR 859, Lille, France; 7 Assistance Publique-Hôpitaux de Marseille, CHU la Timone-Adulte, Marseille, France; 8 Hospices Civils de Lyon, Hôpital Edouard Herriot, Service d’Hygiène, Epidémiologie et Prévention, Lyon, France; Université de Lyon; Université Lyon 1; CNRS, UMR 5558, Laboratoire de Biométrie et Biologie Evolutive, Lyon, France; University Medical Center Rotterdam, The Netherlands

## Abstract

**Background:**

Efforts to provide a valid picture of surgeons’ individual performance evolution should frame their outcomes in relation to what is expected depending on their experience. We derived the learning curve of young thyroidectomy surgeons as a baseline to enable the accurate assessment of their individual outcomes and avoid erroneous conclusions that may derive from more traditional approaches.

**Methods:**

Operative time and postoperative recurrent laryngeal nerve palsy of 2006 patients who underwent a thyroidectomy performed by 19 young surgeons in five academic hospitals were monitored from April 2008 to December 2009. The database was randomly divided into training and testing datasets. The training data served to determine the expected performance curve of surgeons during their career and factors influencing outcome variation using generalized estimating equations (GEEs). To simulate prospective monitoring of individual surgeon outcomes, the testing data were plotted on funnel plots and cumulative sum charts (CUSUM). Performance charting methods were utilized to present outcomes adjusted both for patient case-mix and surgeon experience.

**Results:**

Generation of performance curves demonstrated a gradual reduction in operative time from 139 (95% CI, 137 to 141) to 75 (71 to 80) minutes, and from 15.7% (15.1% to 16.3%) to 3.3% (3.0% to 3.6%) regarding the nerve palsy rate. Charts interpretation revealed that a very young surgeon had better outcomes than expected, whereas a more experienced surgeon appeared to be a poor performer given the number of years that he had already spent in practice.

**Conclusions:**

Not considering the initial learning curve of surgeons exposes them to biased measurement and to misinterpretation in assessing their individual performance for thyroidectomy. The performance chart represents a valuable tool to monitor the outcome of surgeons with the expectation to provide safe and efficient care to patients.

## Introduction

The notion of a developmental learning curve is well known in surgery as in many other professional domains [1], [2]. It can be described as a generalized, predictable improvement in performance witnessed with task repetition over time that tends to be rapid initially, with subsequent smaller improvements leading to an eventual plateau phase [3], [4]. Surgeons intuitively perceive that they need time to accumulate experience at the beginning of their careers or when undertaking a new procedure in order to gain expert skills [5]. Learning curve patterns may vary depending on the type of surgical procedure, the choice of outcome measurements [6], and from one surgeon to another [7]. In addition, learning curves may be influenced by a complex interplay of factors including patient case-mix, the surgeon’s previous experience [8] and training [9], or other institutional factors [10].

Although there is a growing interest in tracking individual surgical outcomes, traditional monitoring tools generally fail to consider the gradual learning process of young surgeons. The ability to generate data of sufficient quality and quantity to investigate surgical learning curves on a procedure-specific basis has historically been a laborious and often impossible task. Fortunately, the recent development and implementation of integrated hospital information systems has permitted the consideration of surgeon-specific factors that influence the value of delivered care [11]. The course of performance of an individual or a group of surgeons can be plotted over time. In principle, recently graduated surgeons are assumed to perform surgery less safely and efficiently than more experienced colleagues at the peak of their career [12], [13]. By extension, efforts to provide a valid picture of individual performance evolution among young surgeons for a particular procedure should frame their outcomes in relation to what is expected depending on their length of experience.

Here we propose a new approach to monitoring the outcomes of inexperienced thyroidectomy surgeons that incorporates the learning curve inherent to this procedure. This methodology utilizes the cumulative sum chart (CUSUM) as a graphical monitoring tool. The CUSUM chart was developed by Page in 1954 [14], and has subsequently been applied in a limited fashion to healthcare performance improvement efforts [15]–[17]. A Risk-Adjusted CUSUM chart has been developed to control for variation in patient case-mix over time when monitoring rare adverse events [18]; furthermore, the Learning Curve CUSUM test has been proposed to determine when surgeons reach a predefined level of performance while learning a new procedure [19]. Our effort represents an application of the CUSUM chart and the funnel plot to the elucidation of the natural evolution of surgical prowess in a manner that simultaneously considers procedure-specific, patient-specific and practitioner-specific factors. We derived the learning curve of young thyroidectomy surgeons as a baseline to enable the accurate assessment of their individual outcomes and avoid erroneous conclusions that may derive from more traditional approaches.

## Materials and Methods

### Study Design and Population

We conducted a prospective, cross-sectional study between April 1, 2008, and December 31, 2009, in five high-volume referral centres in France [13]. From the 28 endocrine surgeons performing thyroid surgery in these academic centres, we selected a subset of 19 young surgeons in their first eight years of practice since graduation; all patients who underwent a thyroid procedure performed by one of these surgeons were eligible for inclusion during a one-year recruitment period. The ethics committee waived the requirement for patient consent. Before surgery, patients received written information about personal data use, and gave verbal consent for sharing their data.

In order to develop the performance chart independently from the data to be monitored, the database was randomly split into training and testing datasets according to surgeon’s identity [20]. The training dataset included procedures performed by 14 surgeons and was used to define the baseline parameters of the performance charts, as well as models for outcomes adjustment. The testing dataset included procedures performed by the 5 other surgeons, each belonging to a different hospital, with the aim of putting the performance charts to the test by checking their application to external data.

### Outcome Measures and Data Collection

Two outcomes were monitored as proxies for young surgeon performance: operative time and postoperative recurrent laryngeal nerve palsy. Operative time was measured in minutes and defined as the total duration from skin incision to closure of the wound. All procedures performed in the participating hospitals were eligible for the operative time analysis. Systematic screening for postoperative recurrent laryngeal nerve palsy was based on the objective evaluation of vocal cord mobility using laryngoscopy within 48 hours following every thyroid procedure [21]. Exclusion criteria for nerve palsy analysis included pre-existing nerve palsy before the intervention, previous thyroid surgery with unknown pre-existing nerve palsy status, and voluntary resection of nerves during intervention due to invasive carcinoma. Additionally, all the procedures performed in one of the participating hospitals were not included in the nerve palsy analysis because in this institution vocal cord mobility was evaluated by laryngoscopy only in cases of postoperative voice alterations.

After each thyroidectomy, a patient report form was completed by the attending surgeon, including items about surgical indication and procedure, as well as the surgeon’s identity and the presence of a more experienced supervisory surgeon during the intervention. Research assistants completed data collection using medical records. These data included patient demographics and information on previous thyroid surgeries, thyroid specimen weights, and assessments of vocal cord mobility. The completeness of inclusions was measured in relation to the number of eligible thyroidectomies recorded in the hospital administrative databases. The surgeon’s length of experience was calculated as the number of years she/he had spent in practice since graduation (i.e., the end of residency).

### Performance Curve Modelling and Case-mix Adjustment

We used the training dataset to determine the expected performance curve of surgeons during their career based on a multivariate generalized estimating equation (GEE) regression model, taking into consideration the clustering of patients by surgeon. The operative time or recurrent laryngeal nerve palsy was the outcome of interest, while surgeon’s experience was the predictor and patient’s case-mix (sex, age, body mass index, thyroid disease, type of thyroidectomy, weight of specimen, supervision by experienced surgeon) was considered as a covariate in the final model. After testing various combinations to enter surgeon experience in the models, a logarithmic shape was finally retained because it provided the best fit.^2^ Expected performance curves were drawn versus the number of years since surgeon’s graduation. Model estimates were obtained using the GENMOD procedure in SAS™ 9.2 (SAS Institute Inc., Cary, NC, USA); all tests were 2-tailed, and *p*-values <0.05 were considered significant.

**Table 1:** Subject characteristics.

Variable		
Patient female gender, No. (*%*, N = 2005)		1558 *(77.7)*
Patient age, years, Mean (*SD*, N = 2002)		50.6 *(14.9)*
Patient body mass index, Mean (*SD*, N = 1961)		26.0 *(5.5)*
Thyroid disease, No. (*%*, N = 2006)	Non-toxic solitary nodule	342 *(17.1)*
	Non-toxic multinodular goiter	1049 *(52.3)*
	Hyperthyroidism	209 *(10.4)*
	Graves’ disease	221 *(11.0)*
	Malignant neoplasm	185 *(9.2)*
Thyroid procedure, No. (*%*, N = 2006)	Unilateral lobectomy	352 *(17.6)*
	Subtotal thyroidectomy	8 *(0.4)*
	Total thyroidectomy	1556 *(77.6)*
	Extended thyroidectomy	11 *(0.6)*
	Completion thyroidectomy	79 *(3.9)*
Lymph node dissection, No. (*%*, N = 1923)		168 *(8.7)*
Weight of thyroid specimen, grams, Mean (*SD*, N = 1960)		44.6 *(42.8)*
Supervision by experienced surgeon, No. (*%*, N = 2006)		237 *(11.8)*
Operative time, minutes, Mean (*SD*, N = 1935)		101.1 *(45.0)*
Recurrent Laryngeal Nerve Palsy, No. (*%*, N = 1366)*		87 *(6.4)*

* Exclusion of one participating centre from the recurrent laryngeal nerve palsy analysis.

Surgical outcomes for the testing dataset were further adjusted using model estimates that were previously generated from the training dataset. For each surgical procedure, the expected operative time or probability of recurrent laryngeal nerve palsy was computed by controlling for patient case-mix alone (classical adjustment) or by simultaneously controlling for patient case-mix and surgeon experience (performance adjustment). Corresponding adjusted outcomes at a given year of experience were calculated as the ratio between the observed and the expected outcomes multiplied by the overall mean operative time or recurrent laryngeal nerve palsy rate. Cross-sectional funnel plots displayed surgeon performance as a function of number of operations [22]. Mean operative time and nerve palsy rate of each surgeon was plotted, applying both the classical and performance adjustment methodologies [23]. Limits were set at one (68.3% CI), two (95.5% CI) and three (99.7% CI) standard deviations around the central line to indicate whether a particular surgeon’s performance differed significantly from the overall mean performance. Underperforming surgeons were positioned above the upper limits, while surgeons with unusually good results were below the lower limits.

**Figure 1:**
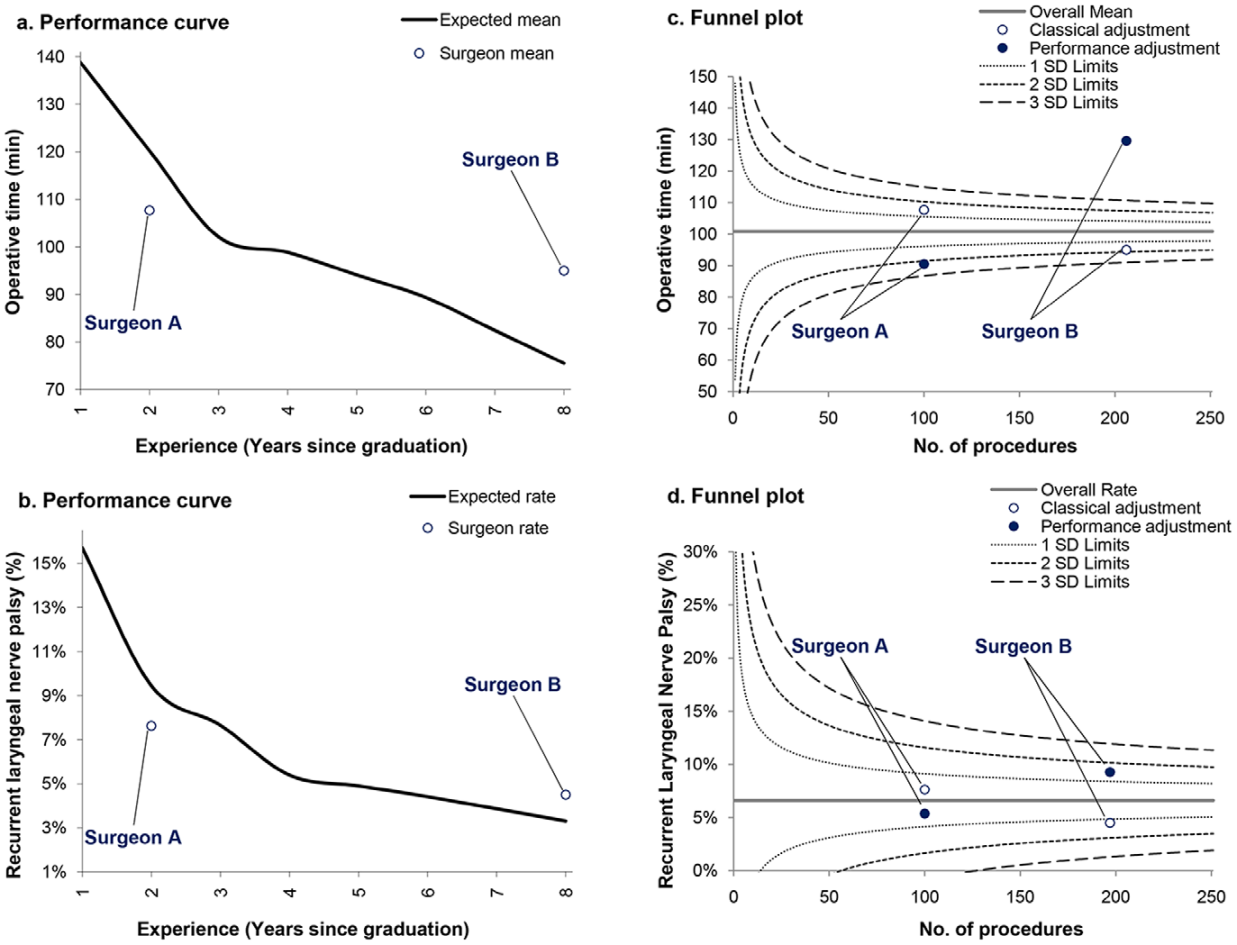
Surgical performance curves and funnel plots for operative time and postoperative complication. Perforamnce curves suggested that mean operative time and recurrent laryngeal nerve palsy rate of surgeon A were lower than expected, while surgeon B performance was poorer than what was expected according to his experience (Fig. 1A and 1B). Funnel plots showed that inverse conclusions could be drawn when interpreting outcomes based on either a classical case-mix adjustment or a comprehensive performance adjustment method (i.e. considering both patient’s case-mix and surgeon’s experience). Surgeon A and B had lower and higher operative time, respectively, than the average based on performance adjustment (Fig. 1C). Similar trends were observed for nerve palsy rates (Fig. 1D).

### Performance Chart Design

Testing data were then plotted on CUSUM charts. Compared to the funnel plot which was based on a single annual assessment of the aggregated performance of every surgeon, the CUSUM chart was updated after each procedure, providing a real-time monitoring of surgeon’s individual performance. The idea was to track surgical outcome to detect as quickly as possible if a small deviation in performance had occurred compared to an expected value. In case of persistent deviation towards a deterioration or improvement in performance, the CUSUM score was supposed to emit a signal by reaching either the upper (h+) or lower (h-) limits, respectively. Conversely, the performance was assumed to be acceptable as long as the CUSUM score remained within the limits.

**Figure 2:**
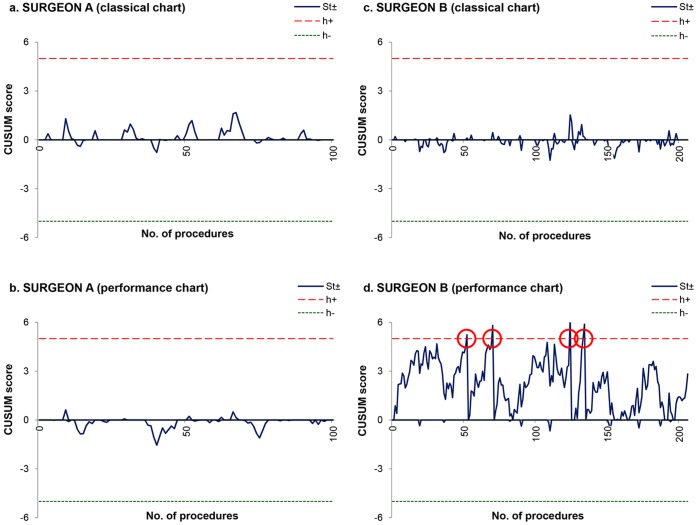
Comparison between classical and performance CUSUM charts for operative time. Operative time was in control for surgeon A on both classical and performance CUSUM charts (Fig. 2A and 2B). CUSUM score for surgeon B was in control according to the classical chart (Fig. 2C), while the upper-sided test of the performance chart signalled four times by crossing the limit at the 52nd, the 70th, the 124th and the 134th procedure (Fig. 2D).

For each surgeon, two types of CUSUM charts were constructed to monitor either operative time or the occurrence of recurrent laryngeal nerve palsy: a classical chart displayed case-mix adjusted surgical outcomes whereas the corresponding performance chart presented outcomes adjusted both for patient case-mix and surgeon experience (Appendix S1). The average run length (ARL) was used to quantify the number of consecutive procedures needed to elicit a signal, either correctly (ARL_1_) or falsely (ARL_0_) on the charts. The monitoring scheme was systematically reset every time the CUSUM plot signalled by crossing the limits [24].

## Results

Of the 3679 eligible thyroidectomies, 3574 (97%) were analyzed in the study period. In accordance with the selection criteria, a total of 2006 procedures were performed by 19 surgeons in their first 8 years of practice. Table 1 shows the main characteristics of surgical cases that were split between the training dataset (14 surgeons having performed 1492 procedures, 74.4%) and the testing dataset (5 surgeons having performed 514 procedures, 25.6%). In the training dataset, half of the surgeons had a length of experience less than 4 years since graduation (min-max, 1 to 8 years), and the median volume of thyroidectomy cases per surgeon was 87 (21 to 324 cases) during the one-year recruitment period. Among the five surgeons who were allocated to the testing dataset, two had performed less than 10 thyroidectomies and another surgeon practiced surgery in the centre excluded from the recurrent laryngeal nerve palsy analysis; we therefore focused on monitoring the outcomes of the two remaining surgeons. Surgeon A was in his second year of practice and had performed 118 procedures during the recruitment period. Surgeon B was more experienced and had started practicing eight years ago with 234 recorded thyroidectomies during the same one-year period. Surgeons A and B declared that they had each performed 200 and 2000 thyroidectomies, respectively, before the beginning of the study.

**Figure 3:**
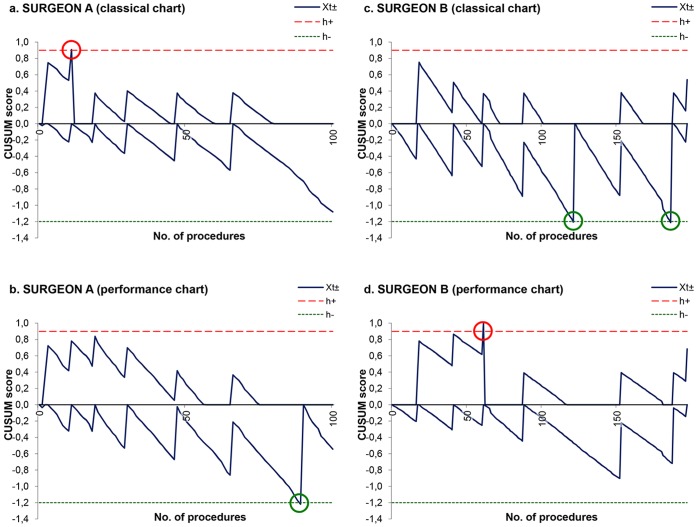
Comparison between classical and performance CUSUM charts for recurrent laryngeal nerve palsy. CUSUM score of recurrent laryngeal nerve palsy for surgeon A crossed the upper limit after 11 procedures on the classical chart (Fig. 3A), whereas it signalled negatively on the performance chart after 89 procedures (Fig. 3B). CUSUM score for surgeon B reached two times the lower limit by the 121st and the 186th procedure on the classical chart (Fig. 3C), whereas it signalled once positively on the performance chart after 61procedures (Fig. 3D).

An inverse relationship was found between surgeon experience and operative time (*p*<0.0001) as well as the frequency of recurrent laryngeal nerve palsy (*p* = 0.002). Generation of performance curves during the first eight years of surgeon practice demonstrated a gradual reduction in operative time from 139 (95% CI, 137 to 141) to 75 (71 to 80) minutes (Figure 1A), and from 15.7% (15.1% to 16.3%) to 3.3% (3.0% to 3.6%) regarding the nerve palsy rate (Figure 1B). Surgeon A appeared to have better outcomes than expected according to his experience, while surgeon B was a poor performer in relation to what was expected. Funnel plots showed dramatic differences in performance measurement between classical and performance adjustment (Figure 1C and 1D). Although surgeon A may have appeared as a poor performer using case-mix adjustment, he proved to have better outcomes than expected after controlling for his length of experience. Conversely, surgeon B had poor outcomes based on performance adjustment instead of being wrongly considered as a high performer based on the classical adjustment method.

Finally, variations in operative time and recurrent laryngeal nerve palsy frequency were monitored on a procedure by procedure basis for surgeons A and B utilizing CUSUM charts (Figures 2 and 3). Surgeon A was interpreted as having poor performance using the classical chart for the occurrence of nerve palsy (Fig. 3A), instead of being regarded as a high performer in accordance with the performance chart that controlled for his lack of experience (Fig. 3B). On the other hand, surgeon B was erroneously identified by the classical charts as having average to good outcomes in terms of operative time (Fig. 2C) and nerve palsy occurrence (Fig. 3C), respectively. After those outcomes were adjusted for surgeon’s experience, the performance chart quickly detected surgeon B as a poor performer (Figs. 2D and 3D).

## Discussion

We have described a new approach to monitoring the individual outcomes of young thyroidectomy surgeons through the development of a data-driven performance curve modelled on procedure-, patient- and practitioner-specific factors. As intuited, our analyses demonstrated substantial improvement in efficiency and safety over time; both operative time and complication occurrence declined gradually with increasing surgeon experience. Indeed, other studies have generally shown that operative time and complication rate decline in a similar fashion with increasing experience and that improvement in performance occurs more rapidly during the early part of a surgeon’s career [7]; specifically, learning curves have been explored in thyroid surgery for the assessment of video-assisted [25], [26], endoscopic [27], and robotically-performed thyroidectomy [28], as well as for intraoperative neuromonitoring [8], [29].

A less anticipated finding, however, was that utilizing these analyses as the backdrop for evaluating individual surgeons’ outcomes revealed marked differences in performance assessment relative to the classical approach [18], [23]. Funnel plots as well as CUSUM chart interpretations diverged between a classical patient case-mix adjustment model and a more comprehensive scheme that also controlled for surgeon experience. The CUSUM performance chart changed the magnitude of indicator variations and the sense of outlier signals; as such, a very young surgeon in the earliest part of his career had better outcomes than expected, whereas a more experienced surgeon was revealed to be a poor performer given the number of years that he had already spent in practice.

In performing this study, we benefitted from a richness of data that enabled us to frame our results with confidence: 1) the multicentre study design included prospective patient recruitment with great thoroughness and reliability in data collection; 2) performance assessment was based on objective and systematic measurement of surgical outcomes; and 3) the train-test approach was expensive in terms of data consumption, but allowed us to validate the chart using an external sample of surgeons [20]. Despite these factors; we acknowledge several limitations to our study. First, the limited number of surgeons in participating French academic hospitals may have produced a potential selection bias. Second, the cross-sectional design of our study captured only a year’s worth of clinical data, and thus included only a small sample of any single surgeon’s experience over the study period. While the construction of an amalgamated performance curve based on cross-sectional data is methodologically sound [12], [30], a more preferable approach would be to monitor the individual performance of a group of surgeons longitudinally over their first decade of practice [6], or to perform a retrospective chart review based on the same design. Third, our choice of postoperative recurrent laryngeal nerve palsy as a proxy for safety may have overstated the long-term complication rate of thyroidectomy, since many such palsies resolve spontaneously with time [31]. Fourth, our study utilized time since graduation as a proxy for attending surgeon experience, as opposed to number of procedures performed. This limitation is a reflection of the cross-sectional nature of the study, as well as the fact that we did not have data concerning the number of thyroidectomy procedures performed by each surgeon prior to the study window.

The implications of these findings warrant further reflection on both the micro and macro strata. On an individual practitioner level, valid performance monitoring is necessary to gain surgeons’ trust and motivate them to rethink the way they are practicing surgery routinely; making performance monitoring tools as rigorous as possible thus facilitates achieving physician buy-in for ongoing performance improvement efforts. On an institutional or higher regulatory level, it has implications for initial training as well as over the long term as part of quality assurance and recertification of practicing specialists. Neglecting to consider the learning process inherent to most surgical procedures predisposes to imperfect measurement and misinterpretation of individual practitioner outcomes [32]. Developmental or disciplinary interventions informed by such erroneous interpretations will arguably be less effective than those based on assessments derived from more accurate assessments that incorporate procedure-, patient- and practitioner-specific factors.

It is also prudent to consider the implications of these findings on efforts to facilitate ongoing surgeon education. In the context of public suspicion regarding suboptimal outcomes, learning a new procedure efficiently is becoming more challenging for young surgeons [33]. Towards this end, utilization of the performance chart to track outcomes in conjunction with simulation approaches [34] may reduce the learning duration by reinforcing self-evaluation and professional mentorship [35]. Compared to classical tools, it allows surgeons to know if they are performing well in relation to what is expected based on their growing experience, with the goal to gradually reduce operative time and complication occurrence. Heads of surgical departments and instructors may also be interested in monitoring the evolution of the individual performance of young surgeons they are mentoring. Performance charts will help them to gauge if a given mentee has gained sufficient expertise level to perform safe surgery. They can detect when a surgeon is experiencing difficulties at some point of her/his career, indicating whether increased intraoperative supervision is warranted. On the other hand, promising surgeons with aptitudes to coach their colleagues will be revealed precociously.

The reproducibility of our methodology must now be tested in a variety of surgical procedures using different performance metrics. Longitudinal outcomes monitoring among large multi-center cohorts of surgeons would be useful in order to accurately predict the performance curve shapes and the acceptable safe standards for particular procedures. In addition, implementing performance chart monitoring of individual surgeons on a prospective basis may help us to further elucidate the factors influencing performance curves, with the expectation of helping surgeons to more rapidly achieve and maintain a high expertise level and optimal outcomes for their patients.

## Supporting Information

Appendix S1 **Methodology for constructing the performance charts.** Detailed formulas for building two-sided cumulative sum charts (CUSUM) charts for continuous or binary data are given.
(DOC)
