# Supplementary material for: Development of a Charting Method to Monitor the Individual Performance of Surgeons at the Beginning of Their Career
Source: PLoS One. 2012 Jul 31;7(7):e41944. doi: 10.1371/journal.pone.0041944 (PMC3409207; doi:10.1371/journal.pone.0041944)
Supplement: Appendix S1 — Methodology for constructing the performance charts. Detailed formulas for building two-sided cumulative sum charts (CUSUM) charts for continuous or binary data are given. (DOC) [file pone.0041944.s001.doc]

**Appendix S1.** Methodology for constructing the performance charts

*CUSUM chart for operative time*

A two-sided CUSUM chart for continuous data displayed the cumulative sums of the deviations in operative time by procedure between the observations and their expected values. The upper-sided CUSUM score $(S_{t}^{+})$ was designed to detect elongation in the mean operative time, whereas the lower-sided CUSUM score $(S_{t}^{-})$ intended for detecting a reduction in the length of procedure based on the following standardized cumulative sums: $S_{t}^{+}=\max\left(0,S_{t-1}^{+}+\frac{x_{t}-\mu}{\sigma}-k\right)$and$S_{t}^{-}=\min\left(0,S_{t-1}^{-}+\frac{x_{t}-\mu}{\sigma}+k\right)$, *t* = 1, 2, 3…, where $x_{t}$ was the adjusted operative time of procedure *t*, with μ and σ as the overall mean and standard deviation of operative time [36], and with the reference value $k=0.5$ [37]. If the CUSUM score $S_{t}^{+}$or $S_{t}^{-}$ reached the predefined limits *h*, out of control signal was emitted due to a significant change in performance. Here, the limits were set at *h* = ±5 to yield ARL_0_ of 465.4 and ARL_1_ of 10.4, 4.0 and 2.6 in detecting shift in operative time equal to one, two and three sigma, respectively [38].

*CUSUM chart for complication*

A two-sided CUSUM chart for binary data was developed to test if a given surgeon deviated positively or negatively from an expected probability of recurrent laryngeal nerve palsy *p*_t_ that was moving for every procedure. Two adjusted CUSUM scores were simultaneously plotted over the course of time, as follows: $X_{t}^{+}=\max(0,X_{t-1}^{+}+W_{t})$and$X_{t}^{-}=\min(0,X_{t-1}^{-}-W_{t})$. $W_{t}$ was the adjusted sample weight of procedure t, such as $Wt=\log\left[\frac{1}{1-p_{t}+ORp_{t}}\right]$ in case of success and $Wt=\log\left[\frac{OR}{1-p_{t}+ORp_{t}}\right]$ if there was a complication,[18] where *OR* was the odds ratio. In this study, the chart was designed to detect either a decreased risk of complication that was equivalent to an *OR* of 0.5 in case of improvement, or an increased risk of complication that was equivalent to an *OR* of 1.5 if the performance deteriorated (i.e. a shift in complication rate under 3.4% or over 9.6% if the expected mean performance was 6.6%). The lower control limit was positioned at -1.2 to yield values of ARL_0_ =145 and ARL_1_=66, whereas we fixed the upper control limit at +0.9 corresponding to ARL_0_ and ARL_1_ values of 138 and 62, respectively.

**References**

**36.** Hawkins DM, Olwell DH. (1998) Cumulative sum charts and charting for quality improvement. New York, NY: Springer.

**37.** Woodall WH, Adams BM. (1993) The statistical design of CUSUM charts. Quality Engineering 5(4): 559-570.

**38.** Lucas JM, Crosier RB. (1982) Fast Initial Response for CUSUM Quality Control Schemes: Give Your CUSUM a Head Start. Technometrics 24: 199–205.
